# *Rhaponticum* genus: a systematic review on traditional uses, phytochemical profiles and pharmacological activities

**DOI:** 10.3389/fphar.2026.1770524

**Published:** 2026-04-22

**Authors:** Wurentuya, Huhezhula, Qirigeer, Shengtao Mei, Sirigunqiqige, Laxinamujila Bai, Ying Xin, Hongyan Hu

**Affiliations:** 1 Mongolian Medicine College of Inner Mongolia Minzu University, Tongliao, China; 2 Research Center of Natural Resources of Chinese Medicinal Materials and Ethnic Medicine, Jiangxi University of Traditional Chinese Medicine, Nanchang, China

**Keywords:** pharmacologi cal, phytochemistry, review, the genus rhaponticum, traditional uses

## Abstract

**Background:**

The genus *Rhaponticum* (Asteraceae/Compositae) comprises 24 species worldwide and has long been utilized in traditional medicine for clearing heat, detoxification, relieving intestinal colic, and treating rheumatoid arthritis, neurasthenia, kidney deficiency, lumbago, and gastrointestinal disorders. Modern pharmacological studies have revealed its diverse bioactivities, including antihypertensive, hypolipidemic, immunomodulatory, neuroprotective, antitumor, hepatoprotective, cardioprotective, anti-inflammatory and anti-influenza effects. However, a comprehensive systematic review integrating its traditional uses, phytochemical metabolites and pharmacological activities is still lacking, which hinders its further development and rational utilization.

**Purpose:**

This study aims to fill the existing research gap by systematically collating and summarizing the traditional medicinal applications, phytochemical compositions, and proven pharmacological activities of *Rhaponticum* species, thereby laying a solid theoretical foundation for the subsequent development, utilization, and in-depth research of this genus.

**Objective:**

This review aims to systematically summarize the traditional applications, phytochemical profiles, and pharmacological activities of *Rhaponticum* species, provide a theoretical basis for their future development and utilization, and highlight the necessity of further investigations into this valuable genus.

**Methods:**

This study was primarily conducted through comprehensive literature search and screening. The retrieval sources included ethnobotanical textbooks, peer-reviewed journals, and scientific databases such as PubMed, Web of Science, Scifinder, and Google Scholar. The search terms encompassed the genus *Rhaponticum*, its representative species (e.g., *Rhaponticum uniflorum, Rhaponticum carthamoides*). Literature screening was based solely on relevance to the focus of the review, with the past 4 decades.

**Results:**

*Rhaponticum* species have a centuries-old history of folk medicinal use, with different ethnic groups utilizing various medicinal parts to treat diverse diseases. A total of 217 metabolites have been isolated and identified from Rhaponticum, covering steroids, flavonoids, sesquiterpenoids, thiophenes, triterpenoids, and other classes. Consistent with traditional uses, modern pharmacological studies have confirmed their antihypertensive, hypolipidemic, antitumor, neuroprotective, anti-inflammatory, cardioprotective, hepatoprotective, antimicrobial, and anti-influenza activities.

**Conclusion:**

Despite 24 documented *Rhaponticum* species, research has predominantly focused on a limited number of Asian species. The material basis, mechanism of action, and therapeutic efficacy of many species remain unclear, and systematic studies on the bioactive metabolites, pharmacological effects, and toxicological profiles of understudied species are insufficient. Given its traditional medicinal value and proven pharmacological activities, further investigations into understudied species, clarification of the molecular mechanisms of core bioactivities, and supplementation of toxicological data are warranted. This review systematically collates the traditional applications, phytochemical compositions, and modern pharmacological value of *Rhaponticum*, providing a valuable reference for its further development and rational utilization.

## Introduction

The genus *Rhaponticum* belongs to the family Asteraceae (formerly Compositae). Plants of this genus are mainly distributed in temperate Eurasian regions worldwide, extensively across Asia, Europe and Africa. According to the Flora Reipublicae Popularis Sinicae, 24 species of the genus *Rhaponticum* have been confirmed globally (Editorial Committee of Flora of China, 1987), among which the eight species involved in the present study are mainly distributed in Asia (China) and Europe (Russia).

For a long time, plants of the genus *Rhaponticum* have played an important role in traditional medicine. They are commonly used for clearing heat and detoxifying, and exert alleviating effects on various ailments including mastitis, intestinal colic, rheumatoid arthritis, neurasthenia, lumbago due to kidney deficiency, and gastrointestinal disorders (Jia and Zhang, 2016; Shakeri et al., 2018; Zughdani et al., 2020). Owing to their valuable ethnomedicinal properties, the genus *Rhaponticum* has gradually attracted extensive attention from the scientific community. To date, researchers have isolated and identified 217 metabolites from the 8 *Rhaponticum* species covered in this study, encompassing major chemical classes such as steroids, flavonoids, terpenoids, and phenylpropanoids. Meanwhile, modern pharmacological studies have further validated the diverse biological activities of this genus, including antihypertensive, lipid-regulating, immunomodulatory, antitumor, hepatoprotective, cardioprotective, anti-inflammatory and anti-influenza effects (Lamer-Zarawska et al., 1996; Wang et al., 2012; Shakhmurova et al., 2010; Skala et al., 2016a; Liu et al., 2020a; Liu et al., 2020; Ge et al., 2021; Hu et al., 2022), providing scientific support for its traditional medicinal value.

Although research on *Rhaponticum* plants has achieved certain progress, obvious limitations still exist. Among the 24 species worldwide, only eight have been reported with studies on chemical metabolites and pharmacology, and research efforts are concentrated on a few Asian species such as *R. uniflorum* and *R. carthamoides*, while the other 16 species remain poorly investigated. Of the identified metabolites, more than 40% lack *in vitro* or *in vivo* activity data, and their pharmacodynamic material basis has not been fully elucidated. In addition, some traditional medicinal efficacies (e.g., treating joint diseases and tonifying the kidney) have not been supported by modern experimental evidence, and studies on the related mechanisms are scattered rather than forming a systematic framework.

Accordingly, this paper systematically reviews and analyzes the research advances in ethnomedicinal uses, chemical metabolites and pharmacological activities of *Rhaponticum* plants over the past 4 decades. It aims to summarize the existing research findings, clarify the research gaps, and provide a scientific basis for the rational development and sustainable utilization of this highly promising medicinal genus.

## Traditional uses

An analysis of existing literature reveals that four *Rhaponticum* species are extensively utilized in traditional medicine for heat-clearing, detoxification, and the treatment of various disorders, including mastitis, intestinal colic, rheumatic arthritis, and neurasthenia (Table 1).

**TABLE 1:** Traditional applications of plants in genus *Rhaponticum*.

Plant species	Ethnic group	Vernacular names	Medicinal parts	Medicinal uses	Reference
*R. uniflorum* (L.) DC.	Han	Loulu	Roots	Detumescence; treatment of mastitis	National Pharmacopoeia Committee of the People’s Republic of China (2020)
	Korean ethnic group (Chaoxianzu in China)	Loulu; Hong gao ri zhao le	Flowers; roots	Alleviation of heat-toxin syndrome, malignant ulcers, pruritus, urticaria, mammary carbuncle, scrofula	Jia and Zhang (2016)
	Mongol (Mongolian)	Honggol zhur	Flowers	Treatment of plague, exogenous fever, intestinal colic	Editorial Board of Chinese Materia Medica (1999)
	Evenk	Ai mu chu ha; Da hua ji; He shang tou	Roots	Treatment of mastitis, mumps, scrofula, arthritis, rheumatoid osteoarthralgia, hemorrhoids, alopecia, lumbago	Jia and Zhang (2016)
*R. carthamoides* (Willd.) Iljin	Kazakh (Kasak)	Lu cao Maral root Russian leuzea	Roots	Alleviation of lumbago, impotence, physical weakness, amnesia, kidney deficiency, neurasthenia, premature ejaculation	Jia and Zhang, 2016; Kokoska and Janovska (2009)
*R. repens* (L.) Hidalgo	Not specified	Not stated	Aerial parts	Treatment of stomach ache, fever, and dysentery	Shakeri et al. (2018)
*R.acaule* (L.) DC.	Not specified	Not stated	Aerial parts	Treatment of pulmonary diseases, gastritis, and rheumatism	Zughdani et al., 2020; Batoul et al., 2014

*Rhaponticum uniflorum* (L.) DC. is the most well-documented species in traditional Chinese medicine (TCM). Its root was first recorded in Shennong Bencao Jing (The Divine Farmer’s Materia Medica), the earliest surviving pharmacopoeia in China, which describes its efficacy in treating mastitis, malignant ulcers, hemorrhoids, abscesses, rheumatoid arthritis, and promoting lactation (Ma, 2013). In traditional Mongolian medicine, the flowers of *R. uniflorum* hold significant therapeutic value, as documented in two classic Mongolian medical texts: 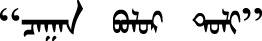 Čagan Bulur Tuli (White Crystal Pearl, 17th century AD) (Yixibalazhuer, 1998) and 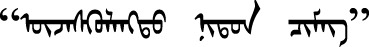 Üjesgülengtü Nidin Čimeg (Essential Compendium of Mongolian Medicine, 19th century AD) (Zhanbuladaoerji, 1998). These texts note that the flowers possess heat-clearing, detoxifying, and exterior-relieving properties, and are clinically employed in treating intestinal colic, epizootic fever, throat abscess, measles, toxic fever, blood heat, acute and chronic heat syndromes, and wound inflammation.

*Rhaponticum carthamoides* (Willd.) Iljin, commonly known as “Xinjiang ginseng” in China, has a long history of use as a stimulant and tonic in the former Soviet republics. Its root and rhizome extracts were formulated into tonic beverages and widely used to alleviate age-related sexual insufficiency, fatigue, impotence, and cardiovascular disorders.

*Rhaponticum repens* (L.) Hidalgo, a traditional medicinal plant in Central Asia, is also recognized as one of the most invasive weed species, exhibiting stronger competitiveness than native weeds in invaded habitats (Dashti et al., 2022). Traditionally, it has been used to treat fever, stomach ache, and dysentery (Shakeri et al., 2018).

In North and Central Tunisia, *Rhaponticum acaule* (L.) DC. is utilized as an aperitif, digestive aid, and tonic, and is employed in the treatment of gastric diseases, tuberculosis, and rheumatism (Mosbah et al., 2018). Additionally, this species has been identified as a potential source of natural herbicidal agents (Mosbah et al., 2020).

## Phytochemistry

### Steroid

**FIGURE 1:**
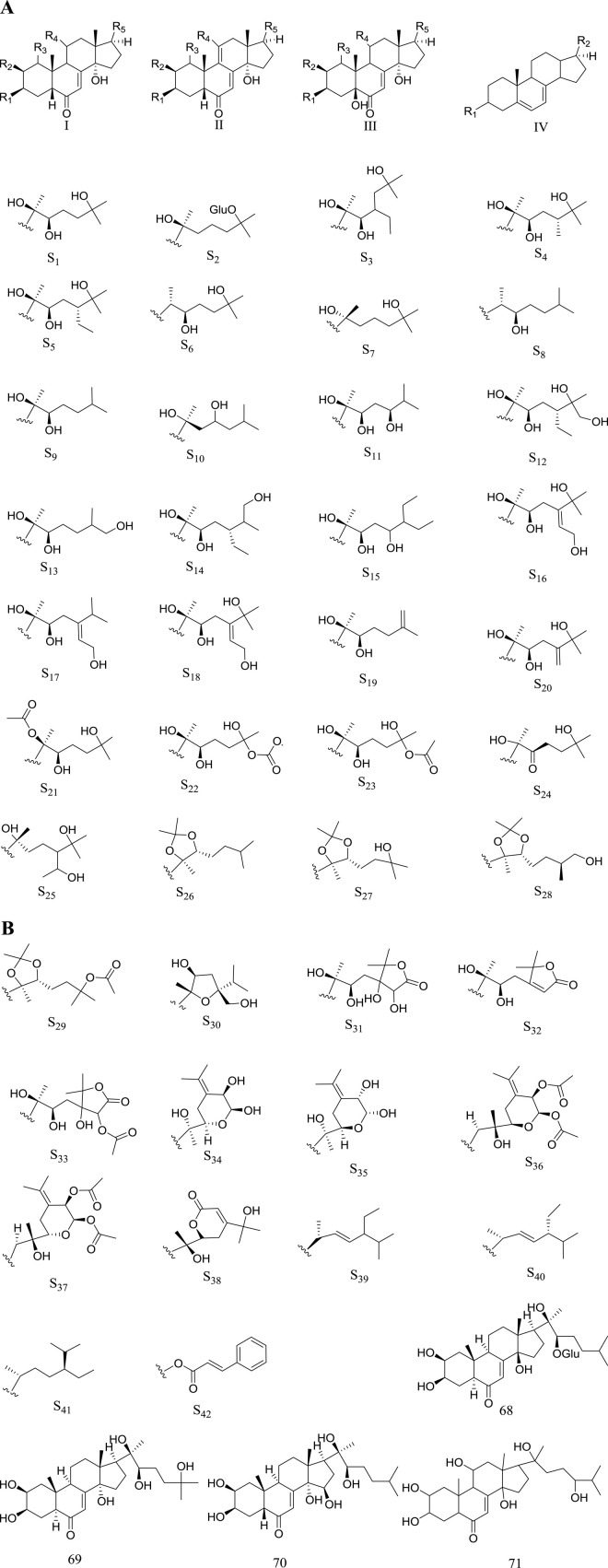
**(A,B)** Steroids d metabolites of *Rhaponticum* (I-IV) The parent nuclei. (S_1_-S_38_) Substituents of the steroid metabolites of *Rhaponticum*. (68–71) Steroids d metabolites 68–71.

Steroid is the main characteristic structural type in the plants of genus *Rhaponticum.* It carried a mother nucleus with a four rings structure containing 21 carbons. The steroids of genus *Rhaponticum* involved sterones and sterols, their difference lies in the fact that sterones have a keto carbonyl at the C-6, while the sterols do not. To this day, 71 steroid metabolites (**1**–**71**) have been obtained from the plants of *Rhaponticum.* The names and structures are shown in the (Table 2) and (Figure 1).

**TABLE 2:** Steroids of the genus *Rhaponticum*.

No	Metabolites	Parent nucleus	Substituents	Source	References
1	Turkesterone	I	R_1_ = R_2_ = β-OH R_3_ = H R_4_ = α-OH R_5_ = S_1_	Underground parts of *R. acaule,* leaves of *R.uniflorum*	Zughdani et al., 2020 Olennikov, 2018a; Budesinsky et al., 2008
2	Acaulesterone	I	R_1_ = R_2_ = β-OH R_3_ = H R_4_ = α-OH R_5_ = S_31_	Underground parts of *R. acaule*	Zughdani et al. (2020)
3	Rhapocasterone A	I	R_1_ = R_2_ = β-OH R_3_ = H R_4_ = α-OH R_5_ = S_34_	Underground parts of *R. acaule*	Zughdani et al. (2020)
4	Rhapocasterone A pentaacetate	I	R_1_ = R_2_ = β-OH R_3_ = H R_4_ = α-OH R_5_ = S_36_	Underground parts of *R. acaule*	Zughdani et al. (2020)
5	Rhapocasterone B	I	R_1_ = R_2_ = β-OH R_3_ = H R_4_ = α-OH R_5_ = S_35_	Underground parts of *R. acaule*	Zughdani et al. (2020)
6	Rhapocasterone B Pentaacetate	I	R_1_ = R_2_ = β-OH R_3_ = H R_4_ = α-OH R_5_ = S_37_	Underground parts of *R. acaule*	Zughdani et al. (2020)
7	Rhapontisterone C	I	R_1_ = R_2_ = β-OH R_3_ = H R_4_ = α-OH R_5_ = S_3_	Roots of *R. uniflorum* Seeds of *R. carthamoides*	Cheng et al., 2002a; Baltaev, 1992
8	Carthamosterone B	I	R_1_ = R_2_ = β-OH R_3_ = R_4_ = H R_5_ = S_22_	Roots of *R. carthamoides*	Ramazanov et al. (1997b)
9	Integristerone A	I	R_1_ = R_2_ = R_3_ = β-OH R_4_ = H R_5_ = S_1_	Leaves of *R. uniflorum*	Olennikov, 2018b; Budesinsky et al., 2008
10	Rhapontisterone	I	R_1_ = R_2_ = β-OH R_3_ = H R_4_ = α-OH R_5_ = S_11_	Leaves, roots of *R. uniflorum*	Olennikov, 2018a; Olennikov, 2018b; Gao et al., 1991
11	Rhaponticum	I	R_1_ = R_2_ = β-OH R_3_ = R_4_ = H R_5_ = S_15_	Roots of *R. uniflorum*	Deng et al. (2000)
12	(24Z)-29-hydroxy-24(28)-dehydromakisterone C	I	R_1_ = R_2_ = β-OH R_3_ = R_4_ = H R_5_ = S_18_	Leaves of *R.uniflorum*	Olennikov (2018b)
13	Inokosterone	I	R_1_ = R_2_ = β-OH R_3_ = R_4_ = H R_5_ = S_13_	Leaves of *R. uniflorum*	Olennikov, 2018b; Budesinsky et al., 2008
14	Makisterone A	I	R_1_ = R_2_ = β-OH R_3_ = R_4_ = H R_5_ = S_4_	Roots, seeds of *R. carthamoides*	Vokac et al., 2002; Baltaev, 1992; Pis et al., 1994
15	Makisterone C	I	R_1_ = R_2_ = β-OH R_3_ = R_4_ = H R_5_ = S_5_	Leaves of *R. uniflorum* Roots of *R. carthamoides*	Olennikov, 2018b; Zhong et al., 2018; Huang et al., 2009; Girault et al., 1988
16	Ponasterone A	I	R_1_ = R_2_ = β-OH R_3_ = R_4_ = H R_5_ = S_9_	Leaves of *R. uniflorum*	Olennikov (2018a)
17	22-oxo-20-hydroxyecdysone	I	R_1_ = R_2_ = β-OH R_3_ = R_4_ = H R_5_ = S_24_	Rootss of *R. carthamoides*	Vokac et al. (2002)
18	20-hydroxyecdysone-2,3-monoacetonide	I	R_1_ = R_2_ = COCH_3_ R_3_ = R_4_ = H R_5_ = S_1_	Rootss of *R. carthamoides*	Vokac et al. (2002)
19	20-hydroxyecdysone-2-O-cinnamate	I	R_1_ = β-OH R_2_ = S_42_ R_3_ = R_4_ = H R_5_ = S_1_	Leaves of *R. uniflorum*	Olennikov (2018b)
20	3-epi-20-hydroxyecdysone	I	R_1_ = α-OH R_2_ = β-OH R_3_ = R_4_ = H R_5_ = S_1_	Roots of *R. carthamoides*	Vokac et al. (2002)
21	20-hydroxyecdysone	I	R_1_ = R_2_ = β-OH R_3_ = R_4_ = H R_5_ = S_1_	Underground parts of *R. acaule*, leaves of *R. uniflorum, R. integrifolium*	Zughdani et al., 2020 Olennikov, 2018b; Zhang et al., 2010; Mamatkhanov et al., 1979; Ramazanov et al., 1997a
22	20-hydroxyecdysone-2-O-acetate	I	R_1_ = β-OH R_2_ = COCH_3_ R_3_ = R_4_ = H R_5_ = S_1_	Leaves of *R.uniflorum*	Olennikov (2018b)
23	20-hydroxyecdysone-3-O-acetate	I	R_1_ = COCH_3_ R_2_ = β-OH R_4_ = R_3_ = H R_5_ = S_1_	Leaves of *R. uniflorum*	Olennikov (2018b)
24	20-hydroxyecdysone-20,22-acetonide-25-acetate	I	R_1_ = R_2_ = β-OH R_3_ = R_4_ = H R_5_ = S_29_	Leaves of *R.uniflorum*	Olennikov (2018b)
25	20-hydroxyecdysone-2,3; 20,22-diacetonide	I	R_1_ = R_2_ = COCH_3_ R_3_ = R_4_ = H R_5_ = S_27_	Leaves of *R. uniflorum*	Olennikov, 2018b; Pis et al., 1994
26	2-deoxy-20-hydroxyecdysone	I	R_1_ = β-OH R_2_ = R_3_ = R_4_ = H R_5_ = S_1_	Leaves of *R. uniflorum*	Olennikov (2018b)
27	25-deoxyecdysone	I	R_1_ = R_2_ = β-OH R_3_ = R_4_ = H R_5_ = S_8_	Leaves of *R. uniflorum*	Olennikov (2018b)
28	Inokosterone-20,22- acetonide	I	R_1_ = R_2_ = R_3_ = β-OH R_4_ = H R_5_ = S_28_	Leaves of *R. uniflorum*	Olennikov, 2018b; Zhong et al., 2018
29	Ajugasterone C	I	R_1_ = R_2_ = β-OH R_3_ = H R_4_ = α-OH R_5_ = S_9_	Leaves of *R. uniflorum* Roots of *R. carthamoides*	Olennikov, 2018b; Zhong et al., 2018; Zhong et al., 2018; Szendrei et al., 1988; Girault et al., 1988
30	Ajugasterone C-20,22-acetonide	I	R_1_ = R_2_ = β-OH R_3_ = H R_4_ = α-OH R_5_ = S_26_	Leaves of *R. uniflorum*	Olennikov (2018b)
31	Ajugasterone C-2,3; 20,22- diacetonide	I	R_1_ = R_2_ = COCH_3_ R_3_ = H R_4_ = α-OH R_5_ = S_26_	Leaves, roots of *R. uniflorum*	Olennikov, 2018b; Zhang andWang, 2001
32	α-ecdysone	I	R_1_ = R_2_ = β-OH R_3_ = R_4_ = H R_5_ = S_6_	Roots of *R. carthamoides* *R. integrifolium*	Budesinsky et al. (2008)
33	Ecdysterone-3-O-β-D-glucopyranoside	I	R_1_ = O-β-D-Glu R_2_ = β-OH R_3_ = R_4_ = H R_5_ = S_6_	Roots of *R*. *uniflorum*	Li et al. (2000)
34	Ecdysterone-25-O-β-D- glucopyranoside	I	R_1_ = R_2_ = β-OH R_3_ = R_4_ = H R_5_ = S_2_	Roots of *R. uniflorum*	Li et al. (2000)
35	Integristerone A 20,22-acetonide	I	R_1_ = R_2_ = R_3_ = β-OH R_4_ = H R_5_ = S_27_	Roots of *R. carthamoides*	Budesinsky et al. (2008)
36	26-hydroxymakisterone C	I	R_1_ = R_2_ = β-OH R_3_ = R_4_ = H R_5_ = S_12_	Roots of *R. carthamoides*	Budesinsky et al. (2008)
37	22-deoxy-28-hydroxymakisterone C	I	R_1_ = R_2_ = β-OH R_3_ = R_4_ = H R_5_ = S_25_	Roots of *R. carthamoides*	Budesinsky et al. (2008)
38	1β-hydroxymakisterone C	I	R_1_ = R_2_ = R_3_ = β-OH R_4_ = H R_5_ = S_5_	Roots of *R. carthamoides*	Budesinsky et al. (2008)
39	Amarasterone A	I	R_1_ = R_2_ = β-OH R_3_ = R_4_ = H R_5_ = S_14_	Roots of *R. carthamoides*	Budesinsky et al. (2008)
40	Uniflorsterone	I	R_1_ = R_2_ = β-OH R_3_ = α-OH R_4_ = H R_5_ = S_10_	Roots of *R. uniflorum*	Cheng et al. (2002b)
41	Leuzeasterone	I	R_1_ = R_2_ = β-OH R_3_ = R_4_ = H R_5_ = S_38_	Roots of *R. carthamoides*	Vokac et al. (2002)
42	Isovitexirone	I	R_1_ = R_2_ = β-OH R_3_ = H R_4_ = α-OH R_5_ = S_19_	Roots of *R. carthamoides*	Vokac et al., 2002; Pis et al., 1994
43	Taxisterone	I	R_1_ = R_2_ = β-OH R_3_ = R_4_ = H R_5_ = S_7_	Roots of *R. carthamoides*	Vokac et al. (2002)
44	Rubrosterone	I	R_1_ = R_2_ = β-OH R_3_ = R_4_ = H R_5_ = C=O	Roots of *R. carthamoides*	Vokac et al. (2002)
45	Dihydrorubrosterone	I	R_1_ = R_2_ = β-OH R_3_ = R_4_ = H R_5_ = OH	Roots of *R. carthamoides*	Vokac et al. (2002)
46	Poststerone	I	R_1_ = R_2_ = β-OH R_3_ = R_4_ = H R_5_ = COCH_3_	Roots of *R. carthamoides*	Vokac et al. (2002)
47	2,3,20,22-diacetonideajugasterone	I	R_1_ = R_2_ = COCH_3_ R_3_ = H R_4_ = α-OH R_5_ = S_26_	Roots of *R. uniflorum*	Zhang andWang (2001)
48	Viticosterone E	I	R_1_ = R_2_ = β-OH R_3_ = R_4_ = H R_5_ = S_23_	*R. integrifolium* Roots of *R. carthamoide*s	Sagdullaev et al., 1999; Girault et al., 1988
49	24(28)-dehydromakisterone A	I	R_1_ = R_2_ = β-OH R_3_ = R_4_ = H R_5_ = S_20_	*R. integrifolium*	Sagdullaev et al., 1999; Girault et al., 1988; Ramazanov et al., 1997a; Girault et al., 1988
50	24 (24^1^) [Z]-dehydroam arasterone B	I	R_1_ = R_2_ = β-OH R_3_ = R_4_ = H R_5_ = S_17_	Seeds of *R. carthamoides*	Baltayev et al. (1997)
51	Ecdysterone 20,22-monoa etonide	I	R_1_ = R_2_ = β-OH R_3_ = R_4_ = H R_5_ = S_27_	*R. carthamoides*	Ramazanov et al., 1997b; Pis et al., 1994
52	Lesterone	I	R_1_ = R_2_ = α-OH R_3_ = H R_4_ = β-OH R_5_ = S_1_	Seeds of *R. carthamoides*	Borovikova and Baltaev (1999a)
53	CarthamoleusteroneB	I	R_1_ = R_2_ = β-OH R_3_ = H R_4_ = α-OH R_5_ = S_30_	Roots of *R. carthamoides*	Budesinsky et al., 2008; Ramazanov et al., 1997b
54	Rhapontisterone R	I	R_1_ = R_2_ = β-OH R_3_ = α-OH R_4_ = H R_5_ = S_32_	Roots of *R. uniflorum*	Li et al. (2000)
55	2,3,11,22,28-pentaacetyl-acculesterone	I	R_1_ = R_2_ = R_4_ = COCH_3_ R_3_ = H R_5_ = S_32_	Underground parts of *R. acaule*	Zughdani et al. (2020)
56	5-deoxykaladasterone-20,22-monoacetonide	II	R_1_ = R_2_ = β-OH R_3_ = R_4_ = H R_5_ = S_26_	Roots of *R. uniflorum*	Zhang andWang (2001)
57	5- deoxykaladasterone	II	R_1_ = R_2_ = β-OH R_3_ = R_4_ = H R_5_ = S_9_	*R*. *carthamoides*	Szendrei et al. (1988)
58	Polypodine-2-O-cinnamate	III	R_1_ = β-OH R_2_ = S_42_ R_3_ = R_4_ = H R_5_ = S_1_	Leaves of *R. uniflorum*	Olennikov (2018b)
59	Polypodine B-20,22-acetonide	III	R_1_ = R_2_ = β-OH R_3_ = R_4_ = H R_5_ = S_27_	Leaves of *R. uniflorum*	Olennikov, 2018a; Olennikov, 2018b Pis et al. (1994)
60	Polipodine B	III	R_1_ = R_2_ = β-OH R_3_ = R_4_ = H R_5_ = S_1_	Leaves of *R. uniflorum* Roots of *R. carthamoides*	Olennikov, 2018b; Zhong et al., 2018; Zhong et al., 2018; Pis et al., 1994; Girault et al., 1988
61	Carthamosterone A	III	R_1_ = R_2_ = β-OH R_3_ = R_4_ = H R_5_ = S_22_	*R. carthamoides*	Ramazanov et al. (1997b)
62	Rhapisterone D 20-acetate	III	R_1_ = R_2_ = β-OH R_3_ = R_4_ = H R_5_ = S_21_	Seeds of *R. carthamoides*	Borovikova et al. (1999b)
63	Integristerone B	III	R_1_ = R_2_ = R_3_ = β-OH R_4_ = H R_5_ = S_1_	Roots of *R.carthamoides,* R. integrifolium	Vokac et al., 2002; Baltaev et al., 1978
64	RapisteroneD	III	R_1_ = R_2_ = α-OH R_3_ = R_4_ = H R_5_ = S_1_	Seeds of *R.carthamoides*	Baltaev U. A. (1995)
65	Stigmasterol	IV	R_1_ = β-OH R_2_ = S_39_	Roots of *R. uniflorum*	Zhang et al. (2010)
66	Daucosterol	IV	R_1_ = O-D-Glu R_2_ = S_40_	Roots of *R. carthamoides* *R. uniflorum*	Budesinsky et al., 2008; Zhang et al., 2002; Chen and Ding, 1997
67	β-sitosterol	IV	R_1_ = S_41_R_2_ = OH	Roots of *R. uniflorum*	Zhang et al., 2010; Huang et al., 2009; Wang et al., 2001; Chen and Ding, 1997; Guo et al., 1992
68	14-epi-Ponasterone A 22-O-β-D-glucopyranoside	​	​	Roots of *R. carthamoides*	Budesinsky et al. (2008)
69	5-α-20-hydroxyecdysone	​	​	Roots of *R. carthamoides*	Vokac et al. (2002)
70	15-hydroxyponasterone A	​	​	Roots of *R. carthamoides*	Budesinsky et al. (2008)
71	RapisteroneB	​	​	Roots of *R. carthamoides*	Baltaev (1991)

### Flavonoids

**FIGURE 2:**
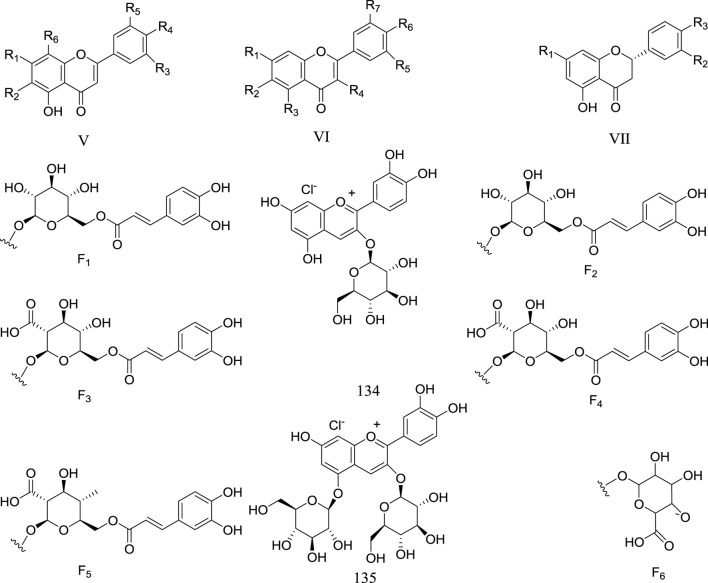
Flavonoid metabolites of *Rhaponticum*. (V–VII) The parent nuclei. (F1–F6) Substituents of the flavonoid metabolites of *Rhaponticum.* (134–135) Flavonoid metabolites 134–135.

Flavonoids are also present in plants of genus *Rhaponticum*, they have various of biological activities, which have attached the attentions of pharmacists. Up to now, 63 flavonoids are gotten from *Rhaponticum*, and can be classified into four structural types: flavonoids (72–112), flavonols (113–130), dihydroflavones (131–133), and anthocyanin (134–135). The names and structures are shown in the (Table 3) and (Figure 2).

**TABLE 3:** Flavonoids of the genus *Rhaponticum*.

No	Compound	Parent nucleus	Substituents	Source	References
72	6-hydroxy luteolin-4′-O-β-D-glucopyranoside	V	R_1_ = F_6_ R_2_ = R_3_ = R_4_ = OH R_5_ = R_6_ = H	Leaves of *R. uniflorum*	Olennikov (2019)
73	Nepetin-3′-O-β-D-glucopyranoside	V	R_1_ = R_3_ = OH R_2_ = OCH_3_ R_5_ = R_6_ = H R_4_ = O-β-D-F_6_	Leaves of *R. uniflorum*	Olennikov and Kashchenko (2019)
74	Lucenin2	V	R_1_ = R_3_ = R_4_ = OH R_2_ = R_6_ = O-β-D-GluR_5_ = H	Leaves of *R. uniflorum*	Olennikov and Kashchenko (2019)
75	Orientin	V	R_1_ = R_3_ = R_4_ = OH R_6_ = O-β-D-Glu R_2_ = R_5_ = H	Leaves of *R. uniflorum*	Olennikov and Kashchenko (2019)
76	Isoorientin	V	R_1_ = R_3_ = R_4_ = OH R_2_ = O-β-D-Glu R_5_ = R_6_ = H	Leaves of *R. uniflorum*	Olennikov and Kashchenko (2019)
77	Vitexin	V	R_1_ = R_4_ = OH R_2_ = R_3_ = R_5_ = OH R_6_ = O-β-D-Glu	Leaves of *R. uniflorum*	Olennikov and Kashchenko (2019)
78	Isovitexin	V	R_1_ = R_3_ = OH R_2_ = O-β-D-Glu R_4_ = R_5_ = R_6_ = H	Leaves of *R. uniflorum*	Olennikov and Kashchenko (2019)
79	6,8-dihydroxyluteolin-7- O-glucoside	V	R_1_ = O-β-D-Glu R_3_ = R_4_ = R_6_ = OH R_2_ = R_5_ = H	Leaves of *R. uniflorum*	Olennikov and Kashchenko (2019)
80	6-hydroxyluteolin-7-O-rutinoside	V	R_1_ = R_3_ = R_4_ = OH R_2_ = O-Glu-3″-Rha R_5_ = R_6_ = H	Leaves of *R. uniflorum*	Olennikov and Kashchenko (2019)
81	6-hydroxyluteolin-7-O-glucoside	V	R_1_ = R_3_ = R_4_ = OH R_2_ = O-β-D-Glu R_5_ = R_6_ = H	Leaves of *R. uniflorum*	Olennikov and Kashchenko (2019)
82	Nepetin-7-O-rutinoside	V	R_1_ = O-β-D-Glu-3″-O -α-L-Rha R_2_ = OCH_3_ R_3_ = R_4_ = OH R_5_ = R_6_ = H	Leaves of *R. uniflorum*	Olennikov and Kashchenko (2019)
83	Nepetin-7-O-glucoside	V	R_1_ = O-β-D-Glu R_2_ = OCH_3_ R_3_ = R_4_ = OH R_5_ = R_6_ = H	Leaves of *R. uniflorum*	Olennikov and Kashchenko (2019)
84	Luteolin-7-O-rutinoside	V	R_1_ = O-Glu-3″-Rha R_2_ = R_5_ = R_6=_H R_3_ = R_4_ = OH	Leaves of *R. uniflorum*	Olennikov and Kashchenko (2019)
85	Luteolin-7-O-glucoside	V	R_1_ = O-β-D-Glu R_2_ = R_5_ = R_6=_H R_3_ = R_4_ = OH	Leaves of *R. uniflorum*	Olennikov and Kashchenko (2019)
86	Apigenin-7-O-glucoside (cosmosiin)	V	R_1_ = O-β-D-Glu R_2_ = R_3_ = R_5_ = R_6_ = H R_4_ = OH	Leaves of *R. uniflorum*	Olennikov and Kashchenko (2019)
87	Nepetin-4′-O-glucoside	V	R_1_ = OCH_3_ R_2_ = R_3_ = R_6_ = OH R_5_ = H R_4_ = O-β-D-Glu	Leaves of *R. uniflorum*	Olennikov and Kashchenko (2019)
88	Luteolin-4′-O-glucoside	V	R_1_ = R_3_ = OH R_2_ = R_5_ = R_6_ = H R_4_ = O-β-D-Glu	Leaves of *R. uniflorum*	Olennikov and Kashchenko (2019)
89	Luteolin-3′-O-glucoside	V	R_1_ = R_4_ = OH R_2_ = R_5_ = R_6_ = H R_3_ = O-β-D-Glu	Leaves of *R. uniflorum*	Olennikov and Kashchenko (2019)
90	Luteolin-7-O-glucuronide	V	R_1_ = F_1_ R_2_ = R_5_ = R_6_ = H R_3_ = R_4_ = OH	Leaves of *R. uniflorum*	Olennikov and Kashchenko (2019)
91	Apigenin-7-O glucuronide	V	R_2_ = R_3_ = R_5_ = R_6_ = H R_1_ = F_1_ R_4_ = OH	Leaves of *R. uniflorum*	Olennikov and Kashchenko (2019)
92	6-hydroxyluteolin-7-O-(6″-O-caffeoyl) glucoside	V	R_1_ = F_1_ R_2_ = R_4_ = OH R_3_ = R_5_ = R_6_ = H	Leaves of *R. uniflorum*	Olennikov and Kashchenko (2019)
93	Luteolin-7-O-(6″-O-caffeoyl) glucoside	V	R_1_ = F_1_ R_4_ = OH R_2_ = R_3_ = R_5_ = R_6_ = H	Leaves of *R. uniflorum*	Olennikov and Kashchenko (2019)
94	Luteolin-7-O-(6″-O-cinnamoyl) glucoside	V	R_1_ = F_2_ R_2_ = R_4_ = OH R_3_ = R_5_ = R_6_ = H	Leaves of *R. uniflorum*	Olennikov and Kashchenko (2019)
95	6-hydroxyluteolin	V	R_1_ = R_2_ = R_4_ = OH R_3_ = R_5_ = R_6_ = H	Leaves of *R. uniflorum*	Olennikov and Kashchenko (2019)
96	Nepetin	V	R_1_ = R_3_ = R_4_ = OH R_2_ = OCH_3_ R_5_ = R_6_ = H	Leaves of *R. uniflorum*	Olennikov and Kashchenko (2019)
97	5,6,7,3′-tetrahydroxy-4′- methoxyflavone	V	R_1_ = R_2_ = R_3_ = OH R_5_ = R_6_ = H R_4_ = OCH_3_	Leaves, flowers of *R. uniflorum*	Olennikov and Kashchenko 2019; Wu and Bai, 2014
98	Nodifloretin	V	R_1_ = R_2_ = R_4_ = OH R_5_ = R_6_ = H R_3_ = OCH_3_	Leaves of *R. uniflorum*, roots of *R. carthamoides*	Olennikov and Kashchenko 2019; Koleckar et al., 2008
99	Luteolin	V	R_1_ = R_4_ = OH R_2_ = R_3_ = R_5_ = R_6_ = H	Leaves, flowers of *R. uniflorum*	Olennikov and Kashchenko 2019; Wu and Bai, 2014
100	Hispidulin	V	R_1_ = R_4_ = OH R_2_ = OCH_3_ R_3_ = R_5_ = R_6_ = H	Leaves of *R. uniflorum, R. repens*	Olennikov and Kashchenko 2019; Shakeri et al., 2018
101	Diosmetin	V	R_1_ = OCH_3_ R_2_ = R_4_ = OH R_3_ = R_5_ = R_6_ = H	Leaves of *R. uniflorum*	Olennikov and Kashchenko (2019)
102	Chrysoeriol	V	R_1_ = R_4_ = OH R_3_ = OCH_3_ R_2_ = R_5_ = R_6_ = H	Leaves of *R. uniflorum*	Olennikov and Kashchenko (2019)
103	Apigenin	V	R_1_ = R_4_ = OH R_2_ = R_3_ = R_5_ = R_6_ = H	Leaves of *R. uniflorum*	Olennikov and Kashchenko (2019)
104	Luteolin-7-O-α-D-glucopyranoside	V	R_1_ = O-α-D-F_6_ R_2_ = R_5_ = R_6_ = H R_3_ = R_4_ = OH	Flowers of *R. uniflorum*	Wu and Bai (2014)
105	Apigenin-7-O-α-D-glucopyranoside	V	R_1_ = O-α-D-F_6_ R_2_ = R_3_ = R_5_ = R_6_ = H R_4_ = OH	Flowers of *R. uniflorum*	Wu and Bai (2014)
106	Apigenin-7-O-α-D-glucuronide	V	R_1_ = O-α-D-Glu R_2_ = R_3_ = R_5_ = R_6_ = H R_4_ = OH	Flowers of *R. uniflorum*	Wu and Bai (2014)
107	Wogonin	V	R_1_ = OH R_2_ = R_3_ = R_4_ = R_5_ = H R_6_ = OCH_3_	Roots of *R. carthamoides*	Huang et al. (2009)
108	6-hydroxy luteolin-7-O- (2′-O-caffeoyl)-β-D-glucopyranoside	V	R_1_ = F_3_ R_2_ = R_4_ = OH R_3_ = R_5_ = R_6_ = R_3_ = H	Leaves of *R. uniflorum*	Olennikov and Kashchenko (2019)
109	6-hydroxy luteolin-7-O- (6″-O-cinnamoyl)-β-D-glucopyranoside	V	R_1_ = F_4_ R_2_ = R_4_ = OH R_3_ = R_5_ = R_6_ = H	Leaves of *R. uniflorum*	Olennikov and Kashchenko (2019)
110	Nepetin-7-O-(6″-O-caffeoyl)-β-D-glucopyranoside (rhaunoside D)	V	R_1_ = F_3_ R_2_ = OCH_3_ R_4_ = R_6_ = H R_3_ = R_4_ = OH	Leaves of *R. uniflorum*	Olennikov and Kashchenko (2019)
111	Nepetin-7-O-(6″-O-cinnamoyl)-β-D-glucopyranoside (rhaunoside E)	V	R_1_ = F_4_ R_2_ = OCH_3_ R_4_ = R_6_ = H R_3_ = R_4_ = OH	Leaves of *R. uniflorum*	Olennikov and Kashchenko (2019)
112	Luteolin-7-O-(2″-O-caffeoyl)-β-D-glucopyranoside (rhaunoside G)	V	R_1_ = F_5_ R_2_ = OCH_3_ R_4_ = R_6_ = H R_3_ = R_4_ = OH	Leaves of *R. uniflorum*	Olennikov and Kashchenko (2019)
113	3′,4′,5,7-pentahydroxy-6-methoxyflavonol	VI	R_1_ = R_3_ = R_5_ = R_6_ = OCH_3_R_2_ = R_7_ = R_4_ = R_7_ = H	Roots of *R. carthamoides*	Koleckar et al. (2008)
114	3-methoxy-quercetin	VI	R_1_ = R_5_ = R_6_ = OH R_2_ = R_3_ = R_7_ = H R_4_ = OCH_3_	Roots of *R. carthamoides*	Zhong et al., 2018; Dombi et al., 1989
115	6-hydroxyquercetin-7-O-(6″-O-caffeoyl) glucoside	VI	R_1_ = F_1_ R_2_ = R_3_ = R_4_ = R_5_ = R_6_ = OH R_7_ = H	Leaves of *R. uniflorum*	Olennikov and Kashchenko (2019)
116	Quercetin-3-O-α-D-rhamnoside	VI	R_1_ = R_3_ = R_5_ = R_6_ = OH R_4_ = O-α-D-Rha R_2_ = R_7_ = H	Flowers of *R. uniflorum*	Wu and Bai (2014)
117	Rutin	VI	R_1_ = R_5_ = R_6_ = OH R_2_ = R_3_ = R_7_ = H R_4_ = O-Glu-3″-Rha	Flowers of *R. uniflorum*	Wu and Bai (2014)
118	Quercetin-3,3′-dimethylether	VI	R_1_ = R_5_ = OH R_2_ = R_3_ = R_6_ = R_7_ = H R_4_ = R_5_ = OCH_3_	Roots of *R. carthamoides*	Varga et al., 1990; Dombi et al., 1989
119	6-hydroxykaempferol-7-O-(6″-O-acetyl-β-D-glucopyranoside)	VI	R_1_ = F_5_ R_2_ = R_4_ = R_6_ = OH R_3_ = R_5_ = R_7_ = H	Roots of *R. carthamoides*	Koleckar et al. (2008)
120	6-hydroxykaempferol-7-O-glucoside	VI	R_1_ = O-Glu R_2_ = R_4_ = R_6_ = OH R_3_ = R_5_ = R_7_ = H	Leaves of *R. uniflorum*	Olennikov (2019)
121	6-methoxykaempferol-7-Oglucoside	VI	R_1_ = O-Glu R_2_ = OCH_3_ R_4_ = R_6_ = OH R_3_ = R_5_ = R_7_ = H	Leaves of *R. uniflorum*	Olennikov and Kashchenko (2019)
122	6-hydroxykaempferol-7-O-(6″-O-caffeoyl) glucoside	VI	R_1_ = F_1_ R_2_ = OCH_3_ R_4_ = R_6_ = OH R_3_ = R_5_ = R_7_ = R = H	Leaves of *R. uniflorum*	Olennikov and Kashchenko (2019)
123	Quercetin	VI	R_1_ = R_4_ = R_5_ = R_6_ = OH R_2_ = R_3_ = R_7_ = H	Flowers of *R. uniflorum*	Wu and Bai, 2014, Bao et al., 2012, Huang et al., 2009
124	6-methoxyquercetin-7-O-glucoside	VI	R_1_ = O-Glu R_2_ = OCH_3_ R_4_ = R_5_ = R_6_ = OH R_4_ = R_7_ = H	Leaves of *R. uniflorum*	Olennikov and Kashchenko (2019)
125	6-methoxykaempferol	VI	R_1_ = R_4_ = R_6_ = OH R_2_ = OCH_3_ R_3_ = R_5_ = R_7_ = H	*R. carthamoides*	Varga et al. (1990)
126	Kaempferol	VI	R_1_ = R_4_ = R_6_ = OH R_2_ = R_3_ = R_5_ = R_7_ = H	Roots of *R. carthamoides*	Huang et al. (2009)
127	Quercetin 5-O-galactoside	VI	R_1_ = R_4_ = R_5_ = R_6_ = OH R_2_ = R_4_ = R_7_ = OH R_3_ = O-Gal	Roots of *R. carthamoides*	Sharaf et al. (2001)
128	Isorhamnetin 5-O-rhamnoside	VI	R_1_ = R_4_ = R_6_ = OH R_2_ = R_5_ = H R_3_ = O-Rha R_7_ = OCH_3_	Roots of *R. carthamoides*	Sharaf et al. (2001)
129	3-O-methyl-quercetin-5-O-β-D-glucopyranoside	VI	R_1_ = R_5_ = R_6_ = OH R_2_ = R_3_ = R_7_ = H R_3_ = F_6_ R_4_ = OCH_3_	Roots of *R. carthamoides*	Huang et al. (2009)
130	Quercetin-5-O-β-D-glucopyranoside	VI	R_1_ = R_5_ = R_6_ = OH R_2_ = R_4_ = R_5_ = R_7_ = H R_3_ = F_6_	Roots of *R. carthamoides*	Huang et al. (2009)
131	Eriodictyol	VII	R_1_ = R_2_ = R_3_ = OH	Roots of *R carthamoides*	Koleckar et al. (2008)
132	Liquiritin	VII	R_1_ = OH R_3_ = O-Glu R_2_ = H	Roots of *R. carthamoides*	Liu et al. (2003)
133	Eriodictyol-7-β-glucopyranoside	VII	R_1_ = F_6_ R_2_ = R_3_ = OH	Roots of *R. carthamoides*	Koleckar et al. (2008)
134	Chrysanthemin	​	​	Flowers of *R. carthamoides*	Vereskovskii and Chekalinskaya (1978)
135	Cyanin	​	​	Flowers of *R. carthamoides*	Vereskovskii and Chekalinskaya (1978)

### Sesquiterpenes

**FIGURE 3:**
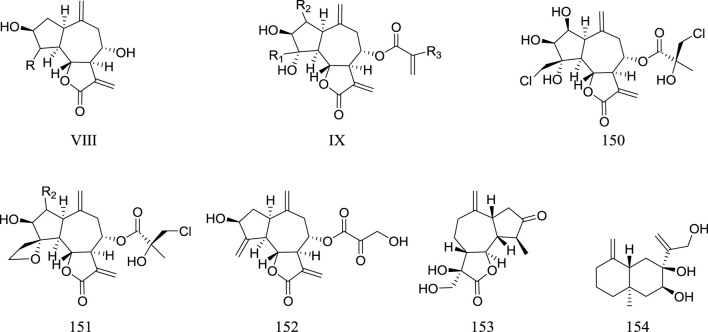
Sesquiterpene metabolites of *Rhaponticum*. (VIII–IX) The parent nuclei. (151–154) Sesquiterpene metabolites 151–154.

Sesquiterpenes are widely existed in nature and have multiple structural types. The sesquiterpenes in the genus *Rhaponticum* mainly belong to the type of guaianolide (**136**–**153**) except the metabolite **154**. Guaianolide is a tricyclic lactone with distinct structural features, one azulene ring is connected to a five membered ring at positions C-1 and C-5, while C-7 forms a five membered gamma lactone ring with C-6 or C-8. The names and structures are shown in the (Table 4) and (Figure 3).

**TABLE 4:** Sesquiterpenes of the genus *Rhaponticum*.

No	Metabolites	Parent nucleus	Substituents	Source	References
136	8-desacylrepin	VIII	R=(CH_2_)_2_OH	Leaves of *R.uniflorum*	Olennikov et al. (2019)
137	Desacylcynaropicrin	VIII	R = CH_2_	Leaves of *R. uniflorum*	Olennikov et al. (2019)
138	15-dechloro-15-hydroxychlorojanerin	IX	R_1_ = CH_2_OH R_2_ = H R_3_ = CH_2_OH	Aerial parts of *R. pulchrum*	Cis et al. (2006)
139	Rhaposerine	IX	R_1_ = CH_2_COOH R_2_ = H R_3_ = CH_3_	*R. serratuloides*	Berdin et al. (1999)
140	Repdiolide	IX	R_1_ = CH_2_ R_2_ = α-OH R_3_ = CH_3_	Aerial parts of *R. pulchrum*	Cis et al. (2006)
141	Cynaropicrin	IX	R_1_ = CH_2_ R_2_ = H R_3_ = CH_2_OH	*R. repens,* aerial parts of *R. pulchrum*	Shakeri et al. (2018)
142	Repensolide	IX	R_1_ = Cl R_2_ = β-OH R_3_ = CH_3_	Aerial parts of *R. pulchrum*	Cis et al. (2006)
143	Aguerin B	IX	R_1_ = CH_2_ R_2_ = H R_3_ = CH_3_	*R. repens*, aerial parts of *R. pulchrum*	Shakeri et al. (2018)
144	Janerin	VIII	R_1_=(CH_2_)_2_OH R_2_ = H R_3_ = CH_2_OH	*R. repens*, aerial parts of *R. pulchrum*	Shakeri et al. (2018)
145	19-deoxyjanerin	VIII	R_1_=(CH_2_)_2_OH R_2_ = H R_3_ = CH_3_	Aerial parts of *R. pulchrum*	Cis et al. (2006)
146	Cebellin G	IX	R_1_ = CH_2_OCOCH_3_ R_2_ = H R_3_ = CH_2_OH	Aerial parts of *R. pulchrum*	Cis et al. (2006)
147	Chlorojanerin	IX	R_1_ = CH_2_Cl R_2_ = H R_3_ = CH_2_OH	Aerial parts of *R. pulchrum*	Cis et al. (2006)
148	Rhaserolide	IX	R_1_ = COOCH_3_ R_2_ = β-OH R_3_ = CH_3_	*R. serratuloides*	Berdin et al. (1999)
149	Cebellin E	IX	R_1_ = CH_2_Cl R_2_ = α-OH R_3_ = CH_3_	*R. repens*	Shakeri et al. (2018)
150	Centaurepensin	​	​	*R.serratuloides*	Berdin et al. (1999)
151	Aeroptilin	​	​	*R. serratuloides*	Berdin et al. (1999)
152	Lactones	​	​	*R. serratuloides*	Berdin et al. (1999)
153	4β,15-dehydro-3-dehydrosolstitialin A	​	​	*R. repens*	Shakeri et al. (2018)
154	Rhaponticol	​	​	*R. uniflorum*	Wei et al. (1997)

### Triterpenoid

**FIGURE 4:**
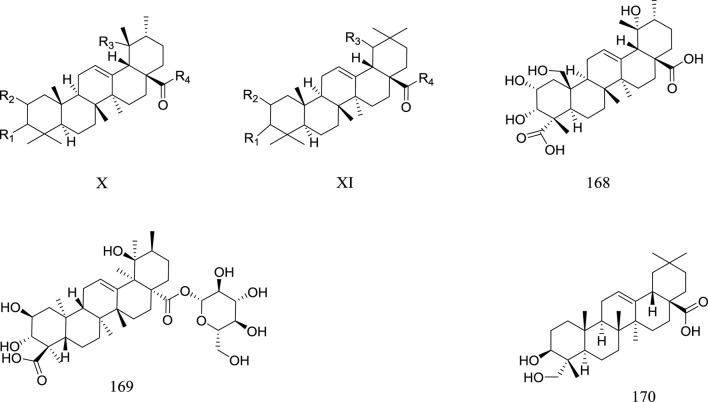
Triterpenoid metabolites of *Rhaponticum*. (X–XI) The parent nuclei. (168–170) Triterpenoid metabolites 168–170.

The triterpenoids in the genus *Rhaponticum* are mainly pentacy clic triterpenoids, including ursane (**155–163, 168–169**) and oleanane (**164–167, 170**). The names and structures are shown in the (Table 5) and (Figure 4).

**TABLE 5:** Triterpenoids of the genus *Rhaponticum*.

No	Metabolites	Parent nucleus	Substituents	Source	References
155	3-Oxo-19α-hydroxyurs-12-en-28-oic acid	X	R_1_ = C=O R_2_ = H R_3_ = α-OH R_4_ = OH	Roots of *R. uniflorum*	Zhang et al., 2002; Zhang and Wang, 2001
156	3-O-α-L-arabinopyranosyl-urs-9(11),12-dien-28-oic acid β-D-glucopyranosyl ester	X	R_1_ = O-α-L-Ara R_2_ = R_3_ = H R_4_ = O-β-D-Glu	Roots of *R. uniflorum*	Zhang et al., 2002; Zhang and Wang, 2001
157	3β-hydroxyurs-12,18(19)-dien-28-oic acid-D-glucopyra nosyl ester	X	R_1_ = β-OH R_2_ = H R_3_ = α-OH R_4_ = O-β-D-Glu	Roots of *R. uniflorum*	Zhang et al. (2002)
158	Ursolic acid	X	R_1_ = β-OH R_2_ = R_3_ = H R_4_ = OH	Roots of *R. carthamoides*	Huang et al., 2009; Zhang et al., 2005
159	Euscaphic acid	X	R_1_ = R_2_ = R_3_ = α-OH R_4_ = OH	Roots of *R. carthamoides*	Huang et al. (2009)
160	3-O-[β-D-glucopyranosyl]-urs-12,19 (29)-dienoic acid-28-O- [β-D- glucop yranosyl] ester	X	R_1_ = R_4_ = O-β-D-Glu R_2_ = H R_3_ = CH_2_	Roots of *R. uniflorum*	Zhang et al. (2009)
161	Pomolic acid	X	R_1_ = β-OH R_2_ = H R_3_ = α-OH R_4_ = OH	Roots of *R. uniflorum*	Zhang and Wang, 2001 Zhang et al., 2002; Zhang et al., 2005
162	Tormentic acid	X	R_1_ = β-OH R_2_ = R_3_ = α-OH R_4_ = OH	Roots of *R. uniflorum*	Zhang et al. (2005)
163	3β -hydroxyurs-12,19(29)-dien −28-oic acid -D-glucop yranosyl ester	X	R_1_ = β-OH R_2_ = H R_3_ = CH_2_ R_4_ = O-β-D-Glu	Roots of *R. uniflorum*	Zhang et al. (2002)
164	rosamultin	XI	R_1_ = β-OH R_2_ = R_3_ = α-OH R_4_ = O-β-D-Glu	Roots of *R. uniflorum*	Zhang et al. (2002)
165	3-O-[β-D-glucopyranosyl]-ilexolic acid-28-O-[β-D-glucopyrano syl] ester	XI	R_1_ = R_4_ = O-β-D-Glu R_2_ = H R_3_ = CH_3_	Roots of *R. uniflorum*	Zhang et al. (2009)
166	Oleanolic acid	XI	R_1_ = α-OH R_2_ = R_3_ = H R_4_ = OH	Roots of *R. uniflorum*	Zhang et al., 2010; Zhang et al., 2005
167	Arjunic acid	XI	R_1_ = β-OH R_2_ = R_3_ = α-OH R_4_ = OH	Roots of *R. uniflorum*	Zhang et al. (2005)
168	2α,3α,19α,25-tetrahydroxyurs-12-en-23,28-dioic acid	​	R_1_ = R_2_ = α-OH R_3_ = OH R_4_ = CH_2_OH	Roots of *R. uniflorum*	Zhang et al. (2002)
169	Sauvissimoside R1	​	R_1_ = β-OH R_2_ = α-OH R_3_ = H R_4_ = O-β-D-Glu	Roots of *R. uniflorum*	Zhang et al. (2002)
170	Hederagenin	​	​	Roots of *R. carthamoides*	Wang et al. (1999)

### Thiophene

**FIGURE 5:**
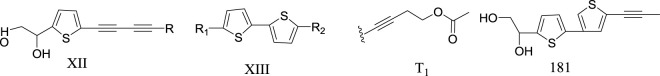
Thiophene metabolites of *Rhaponticum*. (XII–XIII) The parent nuclei. (T1) Substituents of the thiophene metabolites of *Rhaponticum.* (181) Thiophene metabolites 181.

Up to now, 11 thiophene metabolites have been isolated from *Rhaponticum* (Table 6). The main structures of these thiophenes are heterocyclic metabolites and thioethers (Figure 5).

**TABLE 6:** Thiophenes of the genus *Rhaponticum*.

No	Metabolites	Parent nucleus	Substituents	Source	References
171	Rhapontinethiophene B	XII	R = C≡CCH_3_	Roots of *R. uniflorum*	Liu and Guo (2008)
172	2-(pentadiynyl-1,3)-5-(3,4-dihydroxy-butynyl-1) thiophene	XII	R = H	Roots of *R. uniflorum*	Wei et al. (1997)
173	Polyacetylene(E)-1-[5-(hept-5- en1,3-diynyl)-2-thienyl] ethan- 1,2-diol	XII	R = CCH_3_	Roots of *R. carthamoides*	Chobot et al. (2006)
174	Arctinal	XIII	R_1_ = C=O R_2_ = C≡CCH_3_	Roots of *R. uniflorum*	Zhang et al., 2010; Chen and Ding, 1997
175	Arctinone-B	XIII	R_1_ = C=OCH_3_ R_2_ = C≡CCH_3_	Roots of *R. uniflorum*	Zhang et al., 2010; Liu and Guo, 2008; Wei et al., 1997
176	5-(4-acetoxyl-1-butynyl)-2,2′-dithiophene	XIII	R_1_ = H R_2_ = T_1_	Roots of *R. uniflorum*	Zhang et al. (2010)
177	5-methoxyl-2,2′-dithiophene	XIII	R_1_ = H R_2_ = OCH_3_	Roots of *R. uniflorum*	Zhang et al. (2010)
178	7-chloroarctinone B	XIII	R_1_ = OCH_2_Cl R_2_ = C≡CCH_3_	Roots of *R. uniflorum*	Zhang et al., 2010; Liu and Guo, 2008
179	Rhapontiynethiophenes A	XIII	R_1_ = Cl R_2_ = C≡CCH_3_	Roots of *R. uniflorum*	Liu and Guo (2008)
180	Arctic acid	XIII	R_1_ = COOH R_2_ = C≡CCH_3_	Roots of *R. uniflorum*	Liu et al., 2003; Zhang et al., 2002; Chen and Ding, 1997; Wei et al., 1997
181	Arctinol B	​	​	Roots of *R. uniflorum*	Wei et al., 1997; Guo et al., 1992

### Phenypropanoids

**FIGURE 6:**
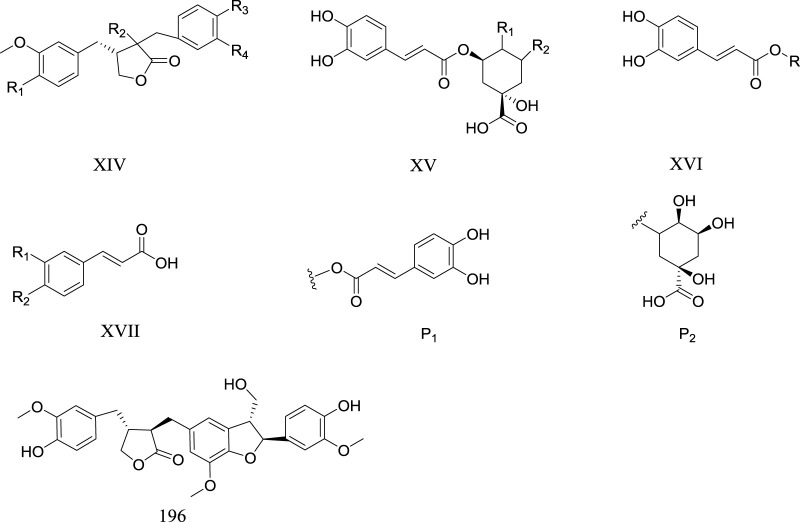
Phenylpropanoid metabolites of *Rhaponticum*. (XIV–XVI) The parent nuclei. (P1) Substituents of the phenylpropanoid metabolites of *Rhaponticum.* (191–193) Phenylpropanoid metabolites 191–193.

Up to now, 15 phenylpropanoid metabolites have been isolated from *Rhaponticum* (Table 7). The phenylpropanoid metabolites of *Rhaponticum* mainly include phenylpropionic acids and lignans (Figure 6).

**TABLE 7:** Phenylpropanoids of the genus *Rhaponticum*.

No	Metabolites	Parent nucleus	Substituents	Source	References
182	Tracheloside	XIV	R_1_ = R_3_ = OCH_3_ R_4_ = O-Glu R_2_ = α-OH R_5_ = H	Seeds of *R. carthamoides*	Harmatha et al. (2007)
183	Trachelogenin	XIV	R_1_ = R_4_ = OCH_3_ R_2_ = α-OH R_3_ = OH R_5_ = H	Seeds of *R. carthamoides*	Harmatha et al. (2007)
184	Carthamogenin	XIV	R_1_ = R_3_ = OCH_3_ R_2_ = R_4_ = OH R_5_ = H	Seeds of *R. carthamoides*	Harmatha et al. (2007)
185	Carthamoside	XIV	R_1_ = R_2_ = R_3_ = OCH_3_ R_4_ = O-Glu R_5_ = H	Seeds of *R. carthamoides*	Harmatha et al. (2007)
186	Isochlorogenic acid A	XV	R_1_ = α-OH R_2_ = α-P_1_	*R. carthamoides*	Vereskovskii and Chekalinskaya (1978)
187	Isochlorogenic acid B	XV	R_1_ = α-P_1_ R_2_ = α-OH	*R*. *carthamoides*	Vereskovskii and Chekalinskaya (1978)
188	Isochlorogenic acid C	XV	R_1_ = β-P_1_ R_2_ = α-OH	*R.carthamoides*	Vereskovskii and Chekalinskaya (1978)
189	Neochlorogenic acid	XV	R = β-P_2_	*R. carthamoides*	Vereskovskii and Chekalinskaya (1978)
190	Chlorogenic acid	XVI	R = α-P_2_	*R. carthamoides*	Vereskovskii and Chekalinskaya (1978)
191	Cinnamic acid	XVII	R_1_ = R_2_ = H	Roots of *R. carthamoides*	Huang et al. (2009)
192	Ferulic acid	XVII	R_1_ = OCH_3_ R_2_ = OH	Roots of *R. carthamoides*	Wang et al. (1999)
193	P-coumaric	XVII	R_1_ = OH R_2_ = H	*R. carthamoides*	Vereskovskii and Chekalinskaya (1978)
194	Caffeic acids	XVII	R_1_ = R_2_ = OH	*R. carthamoides*	Vereskovskii and Chekalinskaya (1978)
195	Syringin	XVII	R_1_ = O-β-D-Glu R_2_ = OCH_3_	Roots of *R. carthamoides* arial parts of *R. pulchrum*	Huang et al., 2009; Cis et al., 2006
196	Lappaol A	​	​	Roots of *R*. *carthamoides*	Huang et al. (2009)

### Other metabolites

**FIGURE 7:**
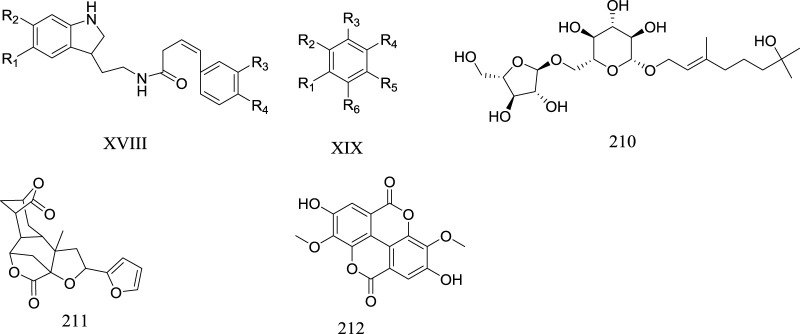
Other metabolites of *Rhaponticum.* (XVII–XX) The parent nuclei. (210–212) Metabolites of *Rhaponticum.*

In addition, 21 other metabolites were isolated from *Rhaponticum*, including alkaloids(197–201), organic acids(202–209, palmitic acid, tetracosanoic acid, and stearate), and disaccharide (210–212, sucrose and maltose) (Table 8) (Figure 7).

**TABLE 8:** Other metabolites of the genus *Rhaponticum*.

No	Metabolites	Parent nucleus	Substituents	Sources	Ref
197	N-(Z)-feruloylserotonin	XVIII	R_1_ = R_3_ = OH R_2_ = H R_4_ = OCH_3_	Seeds of *R. carthamoides*	Harmatha et al., 2007; Yamamotova et al., 2007
198	N-(Z)-isoferuloylserotonin	XVIII	R_1_ = H R_2_ = R_3_ = OH R_4_ = OCH_3_	Seeds of *R. carthamoides*	Harmatha et al. (2007)
199	N-feruloylserotonin	XVIII	R_1_ = R_3_ = H R_2_ = OCH_3_ R_4_ = OH	Roots of *R*. *carthamoides*	Yamamotova et al. (2007)
200	N-(E)-feruloylserotonin	XVIII	R_1_ = R_3_ = H R_2_ = OH R_4_ = OCH_3_	Seeds of *R. carthamoides*	Harmatha et al. (2007)
201	N-(E)-isoferuloylserotonin	XVIII	R_1_ = H R_2_ = OH R_3_ = OCH_3_ R_4_ = OH	Seeds of *R. carthamoides*	Harmatha et al. (2007)
202	Protocatechuic acid	XIX	R_1_ = R_6_ = OH R_2_ = R_3_ = R_5_ = HR_4_ = COOH	Flowers of *R. uniflorum*	Bao et al. (2012)
203	4-hydroxybenzoic acid	XIX	R_1_ = R_2_ = R_4_ = R_5_ = H R_3_ = COOH R_6_ = OH	Roots of *R. carthamoides*	Huang et al. (2009)
204	3,4-dihydroxybenzoic acid	XIX	R_1_ = R_4_ = R_5_ = H R_2_ = R_6_ = OH R_3_ = COOH	Roots of *R. carthamoides*	Huang et al. (2009)
205	3-hydroxy-4-methoxylbenzoic acid	XIX	R_1_ = OCH_3_ R_2_ = OH R_3_ = R_5_ = R_6_ = H R_4_ = COOH	Roots of *R*. *carthamoides*	Huang et al. (2009)
206	2,4-di-hydroxybenzoic acid	XIX	R_1_ = R_5_ = OH R_2_ = R_3_ = R_6_ = H R_4_ = COOH	roots of *R*. *carthamoides*	Huang et al. (2009)
207	*P*-hydroxybenzoic	XIX	R_1_ = R_2_ = R_4_ = R_5_ = H R_3_ = COOH R_6_ = OH	*R. carthamoides*	Vereskovskii and Chekalinskaya (1978)
208	Vanillic acid	XIX	R_1_ = R_3_ = R_6_ = H R_2_ = COOH R_4_ = OCH_3_ R_5_ = OH	*R. carthamoides*	Vereskovskii and Chekalinskaya (1978)
209	Benzyl-O-β-D-glucopyran-oside	XIX	R_1_ = R_2_ = R_4_ = R_5_ = R_6_ = H R_3_ = O-β-D-Glu	Roots of *R. carthamoides*	Huang et al. (2009)
210	(2E)-3,7-dimethyl-1-O-[α-L-arabinofuranosyl-(1→6)-β-D-glucopyranosyl]-oct-2-en-7-ol	​	​	Roots of *R. carthamoides*	Zhong et al. (2018)
211	Diosbulbin B	​	​	Roots of *R. uniflorum*	Liu et al. (2004)
212	3,3,4-tri-(O-methyl) ellagic acid	​	​	Roots of *R. uniflorum*	Zhang et al. (2002)

## Pharmacological effects

Plants of the genus *Rhaponticum* are characterized by their extensive applications in traditional medicine and well-defined pharmacological effects, and their crude botanical drug materials and chemical metabolites have been validated in various *in vitro* and *in vivo* experimental models. Therefore, the pharmacological activities of *Rhaponticum* are summarized in the following subsections.

### Antihypertensive, hypolipidemic and antihyperglycemic effects

In an experiment where a hyperlipidemic mouse model was established by intraperitoneal injection of 75% egg yolk emulsion, the aqueous extract and alcoholic extract of *R. uniflorum* at doses of 150 mg/kg/d and 1350 mg/kg/d reduced the serum TC, LDL-C, and HDL-C levels in mice (Wang et al., 2012).

For rats with experimental myocardial infarction, oral administration of the extract of *R. carthamoide*s at 150 mg/kg combined with quantitative low-intensity exercise for 10 consecutive days improved hematological parameters, reduced whole blood viscosity, decreased red blood cell aggregation, and enhanced red blood cell deformability. In particular, the extract helped increase the hematocrit/blood viscosity ratio, indicating an improvement in the overall blood oxygen transport capacity. Moreover, the combination of the extract and exercise was beneficial in normalizing the concentrations of lactate and pyruvate in the blood of myocardial infarction rats (Plotnikov et al., 2001; Plotnikov et al., 2011).

In addition, integristerone A and ecdysterone isolated from *R. carthamoides* have significantly reduced blood glucose levels in experimental rats induced by adrenaline, alloxan, or glucose administration (Syrov et al., 2012; Dushkin et al., 2014).

### Modulating immune response effects

The pectin metabolite in fresh leaves of *R. carthamoide*s significantly improves phytohemagglutinin (PHA)-induced human lymphocyte proliferation at concentrations up to 150 μg/mL and also significantly enhances the scavenging of oxygen free radicals released by human granulocytes *in vitro* (Lamer-Zarawska et al., 1996). In the experiment, where excessive stress responses were induced by restraining the mice’s backs for 6 h and immune responses were elicited by 2 × 10^7^ sheep red blood cells. The intensity of the immune response was determined by counting the number of antibody-forming cells (AFC) in the spleen using the local hemolysis in gel assay. It was found that the administration of 5 mg/kg of ecdysterone from *R. carthamoides* and 0.5 mg/kg of T-activin had a corrective effect on the negative changes in animals caused by severe stress, and both acted as effective immunomodulators. Compared with the peptide immunomodulator (T-activin), ecdysterone was more effective in preventing and alleviating the consequences of stress and restoring the organism’s immune function (Shakhmurova et al., 2010).

The immune modulating effect of *R*. *uniflorum* extract on cellular and humoral chains of the immune responses was investigated in an immunosupressed mouse model established by oral administration of 50 mg/kg/d azathioprine for five consecutive days. After oral administration of 100 mg/kg of *R*. *uniflorum* extract for 14 consecutive days, the delayed-type hypersensitivity index in the experimental group was 1.9 times higher than that in the control group, and the number of absolute and relative antibody-forming cells increased by 1.9 times and 2.1 times, respectively. Thus, it played an immunomodulatory role in cellular and humoral immune responses (Khobrakova and Tatarinova, 2018).

### Anti-tumor effects

In squamous cell carcinoma (SCC15) cells, the ethyl acetate extract of *R. uniflorum* (RUEA) at 50 mg/mL inhibited cell viability, induced apoptosis, and suppressed cell invasion in a concentration-dependent manner. This was achieved by increasing E-cadherin mRNA and protein expression and decreasing the expression of peroxiredoxin 1 (Prx1), vimentin, and Snail. Moreover, in an oral squamous cell carcinoma (OSCC) xenograft mouse model, RUEA (at doses of 25 mg/kg and 250 mg/kg) significantly inhibited tumor growth (Chen et al., 2017).

Studies have shown that essential oils were extracted from the hairy roots (HR) and soil-grown roots (SGR) of *R*. *carthamoides* by hydrodistillation, while extracts were obtained from the transformed roots (TR) and soil-grown roots (NR) of the plant. GC-MS analysis identified 62 metabolites in each essential oil, with sesquiterpene hydrocarbons as the core metabolites (accounting for 55%–62%) but differences in major metabolites—HR contains cyperene and others, while SGR includes aplotaxene and others. The extracts focused on activities related to human glioma primary cells. Antibacterial experiments with vancomycin, norfloxacin, and amphotericin B as positive controls showed that both essential oils had the best inhibitory effect on bacteria such as *Enterococcus faecalis* (MIC = 125 μg/mL), with stronger antibacterial activity than antifungal activity. Additionally, they could dose-dependently downregulate the expression of inflammatory factors such as IL-1β in LPS-induced astrocytes and reduce ROS levels, showing no cytotoxicity to astrocytes at concentrations of 0–800 μg/mL. In contrast, the TR and NR extracts exhibited cytotoxicity and apoptotic activity, and their effects on cell cycle arrest, mitochondrial membrane potential (ΔΨm), and the expression levels of apoptosis-related genes (Bcl-2, Bax, and p53) were also examined. Overall, these results indicate that HR can serve as an alternative source of essential oil to SGR, and different root extracts of *R*. *carthamoides* have potential application value in multiple fields (Skala et al., 2016a; Skala et al., 2016b).

The 80% methanol extract of *R. carthamoides* transformed roots (Rc TR extract), which is rich in caffeoylquinic acid derivatives, induced glioma cell apoptosis at a dose of 1.5 mg. This was mediated by cleavage and inactivation of PARP1 and inhibition of its synthesis, leading to increased DNA damage, elevated phosphorylated H2A.X levels, and reduced levels of DNA epigenetic markers (e.g., UH1 and DNMT1) (Skala et al., 2018a).

The 80% methanol extracts of Rc TR and *R. carthamoides* soil-grown roots (Rc NR)—both rich in caffeoylquinic acid derivatives—inhibited the viability of three human cancer cell lines in a concentration-dependent manner (0.019–5 mg/mL). Rc TR and Rc NR extracts exhibited similar cytotoxicity against two leukemia cell lines (CCRF-CEM and K-562; IC_50_ = 0.313 mg/mL), which were more sensitive than A549 lung adenocarcinoma cells (IC_50_ = 0.625 mg/mL). Rc TR extract showed stronger inhibitory effects than Rc NR extract. Both extracts also reduced mitochondrial membrane potential in all tested cell lines (2.4-fold in K-562 cells, 1.2-fold in CCRF-CEM and A549 cells) and exhibited genotoxic activity: they increased mtDNA damage in the ND1 region of all cancer cells, mtDNA damage in the ND5 region of leukemia cells (K-562 and CCRF-CEM), and nDNA damage in the TP53 region of leukemia cells (K-562 and CCRF-CEM) (Skala et al., 2018b).

### Neuroprotective effects

The ethanol extract of *R. uniflorum* (at doses of 0.39, 0.78, 1.56, and 3.125 g/kg/d) reduced the number of errors in step-down and avoidance tests in a dose-dependent manner, prolonged error latency, increased superoxide dismutase (SOD) content and lipofuscin production in the brain tissue of D-galactose-induced aging mice (subcutaneously injected with 50 g/L D-galactose at 0.5 mL/day per mouse for 40 consecutive days), and thus improved memory impairment in the mice (Zou et al., 2003).

A 5-day treatment with *R. carthamoides* dry extract (150 mg/kg/day, p.o.; equivalent to 2.7 mg ecdysterone per kg body weight) reduced manifestations of ischemic damage, improved neuronal structure, and restored brain activity (Plotnikov et al., 2005).

In another experiment by Plotnikov et al., 5 days of treatment with the same dose of *R. carthamoides* increased lipid and phospholipid (mainly sphingomyelin and phosphatidylserine) content in the red blood cell membrane and decreased phospholipid proportion in rats with cerebral ischemia. It also improved red blood cell morphology (increased discocyte count, decreased degenerated cell count), thereby alleviating cerebral ischemia symptoms in rats (Plotnikov et al., 2008).

*R. uniflorum* extract exerts multi-dimensional stress-protective and emotional regulatory effects. Pretreatment with 100 mg/kg dose can alleviate Selye’s triad in rats with acute stress, reduce oxidative intensity, exhibit potent scavenging activity *in vitro* against ABTS, DPPH free radicals and superoxide anions, chelate Fe^2+^, and regulate the hypothalamic-pituitary-adrenal (HPA) axis by reducing adrenocorticotropic hormone (ACTH) levels by 40% and corticosterone levels by 24%. This dose exerts antidepressant and anxiolytic effects on rats with chronic stress, shortens the immobility time in the forced swimming test and tail suspension test. Its anxiolytic effect depends on the non-competitive binding site of the GABA-A receptor, which can antagonize bicuculline but is blocked by picrotoxin (Shantanova et al., 2021).

### Anti-inflammatory effects

In a mouse hot-plate test, intraperitoneal injection of *R. uniflorum* volatile oil (0.2 mL/10 g) exerted an analgesic effect at 30 min, peaking at 90 min. In two other mouse pain models (intraperitoneal injection of 0.13 mL/10 g p-benzoquinone or 0.1 mL/10 g 0.1% acetic acid solution), the volatile oil (0.15 mL/10 g) also exhibited analgesic activity (Sun et al., 2003).

*R. uniflorum* raw botanical drug material (1.4 g/kg) and ethanol extract (0.7 g/kg) exhibited anti-inflammatory effects in three models: 1) xylene-induced inflammation in both sides of the right ear in mice (0.1 mL xylene evenly applied), carrageenan-induced inflammation in the subcutaneous tissue of the right hind paw in rats (0.1 mL 1% carrageenan solution injected), and cotton ball-induced granuloma in rats (30 mg sterilized defatted cotton balls with streptomycin and penicillin implanted subcutaneously in the inguinal region). The ethanol extract showed more significant effects. Additionally, 20-hydroxyecdysone (6.25–400 μg/mL), hemislin B glucoside (1.5625–100 μg/mL), luteolin (0.39–25 μg/mL), and gallic acid (1.5625–100 μg/mL) isolated from *R. uniflorum* flowers inhibited lipopolysaccharide (LPS)-induced nitric oxide (NO) release from RAW264.7 cells to varying degrees, with the inhibitory effect being concentration-dependent. Apigenin, quercetin, quercitrin, and afudoside showed strong NO inhibitory activity at 1.5625–400 μg/mL (inhibition >80% at 6.25 μg/mL) and almost completely inhibited NO production at 25–400 μg/mL (Deng, 2015).

The ethanol extract of *R. uniflorum* flowers (100, 200, and 400 mg/kg) significantly improved LPS-induced acute lung injury in mice following 3 days of continuous intragastric administration. This was achieved by reducing NO, interleukin-6 (IL-6), tumor necrosis factor-α (TNF-α), myeloperoxidase (MPO), and SOD levels in bronchoalveolar lavage fluid (BALF), as well as MPO, SOD, catalase (CAT), malondialdehyde (MDA), and glutathione peroxidase (GSH-Px) levels in lung tissue, and improving lung tissue pathological changes. Its anti-inflammatory mechanism may be related to the Nrf2/HO-1 and MAPK/NF-κB signaling pathways, the positive drug dexamethasone (DEX) also exhibits similar protective effects (Zhen et al., 2022).

In 2020, Liu et al. reported that *R. uniflorum* ethanol extract at high doses (200 μg/mL and 400 μg/mL) affected milk component synthesis by regulating the expression of signaling molecules (STAT5, mTOR, SREBP1, AKT1, and GLUT1) in dairy cow mammary epithelial cells (Liu et al., 2020a). In another study, the same research team showed that *R. uniflorum* ethanol extract (25, 50, and 100 μg/mL) upregulated the gene expression of milk anabolic enzymes and proteins in mammary epithelial cells and modulated the synthesis of milk protein, milk fat, and lactose in goat mammary epithelial cells (Liu and Yang, 2020b).

### Antioxidant effects

Researchers found that among different extracts from various parts of *R*. *acaule*, leaf extracts exhibited the highest yield of phenolic metabolites (23.96% ± 2.04%), followed by flower (20.18% ± 2.13%) and root (5.49% ± 1.44%) extracts. Root phenolic extracts showed the strongest DPPH scavenging activity (0.31 ± 0.04 mg/mL), followed by flower tannin extracts (0.35 ± 0.00 mg/mL). Leaf methanol crude extracts had the lowest EC_50_ value (2.61 ± 0.03 mg/mL), while root extracts had a higher EC_50_ value (1.06 ± 0.02 mg/mL) (Bendimerad-Mouttas et al., 2020).

The optimal extraction rate of alkaloids from *R. uniflorum* flowers was achieved using hydrochloric acid-methanol (1:100, v/v) as the solvent and 20 min of ultrasonic treatment. The supernatant alkaloids exhibited superoxide anion scavenging activity at 0.003–0.17 g/L, with activity increasing with concentration (Kang et al., 2015).

The volatile oil yields of *R. carthamoides* hairy roots (HR) and soil-grown roots (SGR) were 0.09% and 0.06% (dry weight, v/w), respectively, and the two essential oils had similar properties. After treating astrocytes with LPS (1 μg/mL) followed by 24-h incubation with HR or SGR essential oils (100 μg/mL), intracellular reactive oxygen species (ROS) levels in LPS-stimulated astrocytes were reduced by approximately 1.5-fold compared with cells treated with LPS alone (Skala et al., 2016a).

Researchers investigates that the pharmacological effects of N-feruloylserotonin (N-f-5HT) isomers isolated from the seeds of *R. carthamoides*. *In vitro* experiments show that they dose-dependently inhibit the oxidative burst of human whole blood and isolated neutrophils, with the most significant effect on PMA-induced stimulation. They can suppress the production of extracellular and intracellular reactive oxygen species (ROS) without affecting cell viability and apoptosis. Mechanistically, N-f-5HT exerts its effect by reducing the phosphorylation of PKC α/β II. Similar to positive drugs such as carvedilol, loratadine, stobadine, and curcumin, it holds promise for research on oxidative stress-related diseases (Nosal et al., 2011).

### Cardioprotective and hepatoprotective effects

The study investigated the protective effect and underlying mechanism of *R. carthamoides* (Rha) on isoproterenol-induced myocardial ischemia in male Sprague-Dawley rats, with Rho as the positive drug control. The results showed that intragastric administration of Rha at different doses improved the phenotypic changes of myocardial ischemia in rats, corrected energy metabolism disorders and alleviated oxidative stress in myocardial tissues and H9c2 cells, while upregulating the expressions of SIRT6 and Nrf2. Functional experiments confirmed that SIRT6 silencing attenuated the regulatory effects of Rha, whereas Nrf2 activation potentiated its effects, indicating that Rha exerts a cardioprotective effect through the SIRT6/Nrf2 signaling pathway (Zheng et al., 2022).

In H9c2 cells treated with 0.75 μM/mL doxorubicin (DOX) for 24 h and a zebrafish DOX-induced cardiotoxicity model (0.75 μM/mL DOX for 120 h), pretreatment with LLF (100, 200, and 400 μg/mL) significantly inhibited DOX-induced H9c2 cell cytotoxicity in a dose-dependent manner. LLF and DEX (dexamethasone, positive control drug) could alleviate DOX (doxorubicin)-induced cardiotoxicity, improve zebrafish heart development, and reduce the excessive production of superoxide dismutase (SOD) and reactive oxygen species (ROS), among which 400 μg/mL (micrograms per milliliter) of LLF exhibited the most significant effect in inhibiting the overproduction of ROS.The mechanism may involve LLF alleviating DOX-induced cardiotoxicity by blocking NF-κB signaling and rebalancing mitochondrial dynamics (Hu et al., 2022).

In a 30 μg/L H_2_O_2_-induced HepG2 cell injury model, *R. uniflorum* aqueous extract (50, 100, and 200 mg/L) inhibited HepG2 cell apoptosis. This was characterized by reduced LDH, alanine transaminase (ALT), and aspartate transaminase (AST) activity in the culture medium; decreased intracellular MDA content; increased SOD activity and GSH content; reduced relative activity of Caspase-3, -8, and -9; downregulated expression of Casp-3 and cytochrome c (Cyto c) in cells and cytoplasm; and decreased levels of p-JNK and nuclear NF-κB proteins. The repair mechanism may be related to the inhibition of JNK activation and NF-κB nuclear translocation (He et al., 2017).

In an experiment investigating the protection against acute liver injury in mice (induced by a single intraperitoneal injection of 500 mg/kg D-galactosamine [GalN]), oral administration of *R. uniflorum* n-butanol extract and aqueous layer extract (200 mg/kg/d) for seven consecutive days significantly reduced serum ALT and AST activity in mice with liver injury. This was achieved by reducing inducible nitric oxide synthase (iNOS) and NO levels in liver tissue, decreasing mitochondrial MDA levels in the liver, and increasing mitochondrial GPx and SOD activity and GSH levels in the liver. *R. uniflorum* extract exerts a protective effect against GalN-induced acute liver injury, which may be related to its antioxidant and anti-inflammatory properties (Jin et al., 2016).

### Other effects

The methanolic extract of *R. acaule* fruit exhibited different inhibition zone diameters against three bacterial strains (*Escherichia coli*, *S. aureus*, and *E. faecalis*) at various dilution concentrations (100%, 75%, 50%, and 25%). *Escherichia coli* and *E. faecalis* were highly sensitive to all extract concentrations, with inhibition diameters ranging from 24 to 45 mm and 9–13 mm, respectively. *Staphylococcus aureus* was highly sensitive to the undiluted extract, with an inhibition diameter of 19 mm (Benabdesselam et al., 2018).

The ethanol extract of *R. carthamoides* leaves showed strong broad-spectrum antibacterial activity. Eukaryotic microorganisms (e.g., *Candida* albicans) were more sensitive to the extract than prokaryotic bacteria. Specifically, a 30-fold dilution (1330 μg/mL) of *R. carthamoides* viscous extract showed the strongest inhibition of *E. coli* ATCC 25922 and *Klebsiella pneumoniae* ATCC 3349. *Bacillus* cereus ATCC 8035 and *Candida* albicans were most sensitive to a 70-fold dilution of the extract. A 70-fold dilution of the viscous extract showed moderate sensitivity against *E. faecalis* ATCC 29212, *Pseudomonas aeruginosa* ATCC 27853, and *Bacillus subtilis* ATCC 6633, while a 105-fold dilution showed moderate sensitivity against *S. aureus* ATCC 25923 (Jurkštienė et al., 2011).

A study on the anti-influenza activity of various extract fractions (30%, 70%, 90% ethanol, ethyl acetate, and aqueous solutions) from LLF showed that the ethyl acetate fraction exhibited the strongest antiviral activity at 1.25 mg/mL, with a virus inhibition rate of 64.59%. The combination of oseltamivir and the ethyl acetate fraction showed a strong synergistic effect (Ge et al., 2021).

The effect of 1 mg/mL *Rhaponticum pulchrum* ethanol extract on the growth of T4 genotype Acanthamoeba spp. Strain Ac55 trophozoites (isolated from a keratitis patient) was tested *in vitro*. The extract exhibited amoebicidal activity in a time- and dose-dependent manner, and its inhibitory potential was primarily dependent on the yield and composition of sesquiterpene lactones (Hadas et al., 2017).

In an acute experiment, rats fed a custom low-protein (10%) diet A04 (30 g/d; protein, antioxidant, and vitamin content mimicking an unfortified self-care diet) underwent 20 ladder-climbing trials per day (10 trials without weight, 10 trials with 50% and 75% body weight loads). Administration of a 50:50 mixture of *R. carthamoides* (Rha) and *Rhodiola rosea* (Rho) at 43.5 mg/kg alleviated bone myofascial pain syndrome (MPS). In a 4-week chronic ladder-climbing experiment (starting with 50% body weight load, increased to 150% body weight load from week 4; 5 times/week, 20 repetitions/trial), the same dose of the Rha-Rho mixture increased rat body weight by 30%, enhanced power output after resistance training, and increased the proportion of type I and type II muscle fibers in the flexor digitorum profundus (Vernus et al., 2020). Additionally, recent studies have indicated that R. uniflorum can also affect hepatic drug‐metabolizing enzymes by regulating the activity and mRNA expression of CYP3A1 in rat hepatocytes (Wu et al., 2007)

## Conclusions and perspectives

*Rhaponticum* is a highly valuable medicinal genus widely used in traditional Chinese medicine and the ethnomedicinal systems of multiple ethnic groups. Studies on this genus have established a solid foundation in phytochemistry and pharmacology: to date, 217 metabolites have been isolated and identified from 8 *Rhaponticum* species. Its major secondary metabolites comprise phytoecdysteroids, steroids, triterpenoids, sesquiterpenoids, flavonoids, organic acids, and thiophenes. Among these, phytoecdysteroids, triterpenoids, guaiane-type sesquiterpenoids, and thiophenes are the most widely distributed and possess important chemotaxonomic significance for the genus.

**FIGURE 8:**
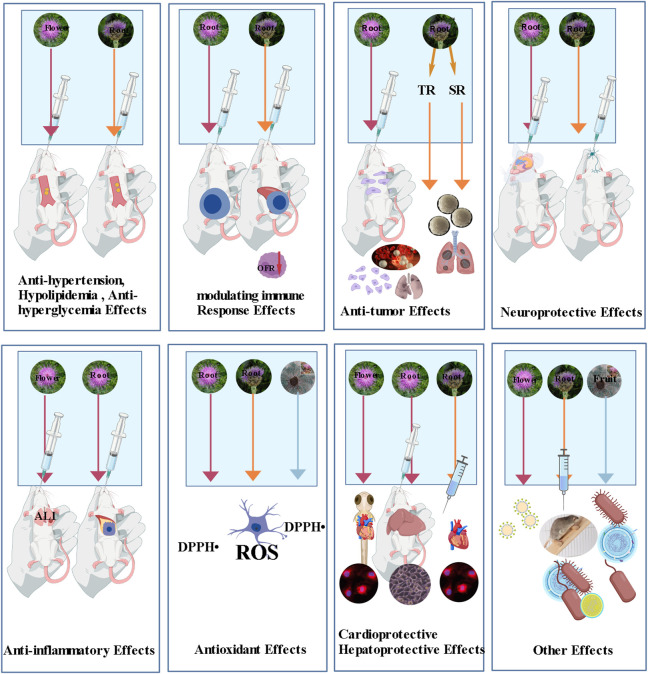
Schematic diagram of the pharmacological effects of *Rhaponticum* plants.

The pharmacological research on plants of the genus *Rhaponticum* is still in the exploratory stage: most of the metabolites isolated from this genus have only been structurally characterized, with their pharmacological activities unreported or poorly understood. Only a few widely distributed metabolites have been confirmed to possess definite biological activities, including 20-hydroxyecdysone, cynaropicrin, ursolic acid, oleanolic acid, as well as common flavonoid aglycones such as luteolin, apigenin and quercetin. In contrast, most of the genus-specific metabolites, including steroid metabolites, acylated flavonoid glycosides, guaiane-type sesquiterpenoids (except cynaropicrin), thiophenes, triterpenoid derivatives, lignans and polyacetylenes, lack systematic pharmacological evaluation. Despite the significant research gaps in individual metabolites, extracts of Rhaponticum plants have been proven to exhibit a broad spectrum of biological activities, such as hypolipidemic, antitumor, antinociceptive, immunomodulatory, anti-inflammatory, antioxidant, neuroprotective and cognitive-enhancing effects (Figure 8). This provides preliminary scientific evidence for its traditional medicinal effects including clearing away heat and treating neurasthenia.

The core characteristic metabolites of *Rhaponticum* also exhibit remarkable species specificity. For instance, Rhapontisterone R1 from *R. uniflorum* and Carthamosterone A/B from *R. carthamoides* are unique metabolites of their respective species. Among them, thiophene metabolites are extremely rare in nature and have thus far only been isolated from *R. uniflorum* and *R. carthamoides*, serving as a key identifier for distinguishing related species of *Rhaponticum* from other plants in the Asteraceae family. Furthermore, daucosterol, β-sitosterol and stigmasterol are common metabolites in all studied species of this genus and can be used as chemotaxonomic markers for related species within *Rhaponticum*. Phytoecdysteroids, triterpenes, guaiane-type sesquiterpenoids and thiophenes are the characteristic secondary metabolites of *Rhaponticum*, which can act as identification markers for differentiating *Rhaponticum* from other related genera of Asteraceae such as Cirsium. Steroids, in turn, serve as a common chemotaxonomic marker for related species within the genus *Rhaponticum*.

Current research on *Rhaponticum* still has three prominent limitations. First, the coverage of studied species is incomplete: among the 24 recognized *Rhaponticum* species worldwide, phytochemical and pharmacological studies have only been conducted on 8, with no relevant reports for the remaining 16. The potential similar bioactivities inferred from phylogenetic relationships remain unexplored and unvalidated. Second, research strategies and methods are overly simplistic: most studies focus only on activity evaluation of crude extracts or *in vitro* metabolite screening targeting single targets, lacking in-depth investigation into the synergistic effects and integrated therapeutic efficacy of multiple active metabolites. Third, the validation of pharmacodynamic substances and traditional efficacies is insufficient: most of the 217 isolated metabolites have not undergone pharmacological testing, the core pharmacodynamic material basis remains unclear, and folk-recorded efficacies such as treating joint disorders and tonifying the kidney lack experimental evidence.

To address these issues, targeted breakthroughs can be made in four aspects in future research. First, expand the research scope by systematically investigating the phytochemical metabolites and pharmacological activities of the 16 understudied species, to fully exploit the medicinal potential of the entire genus. Second, deepen mechanistic research by focusing on pharmacological evaluation of characteristic active metabolites, screening promising monomers or metabolite groups, and clarifying their dose-response relationships, structure-activity relationships, and safety profiles. Third, bridge traditional efficacies with modern validation, experimentally confirm unsubstantiated traditional medicinal values, and improve the scientific understanding of the genus’ medicinal properties. Fourth, explore multi-metabolitesynergistic effects using innovative technical approaches to elucidate the integrated therapeutic interactions among different active metabolites.
